# Leveraging Dental Visits for Systemic Health: Diabetes Screening and Referral Compliance in Periodontitis Patients in Malaysia

**DOI:** 10.3390/jcm14030739

**Published:** 2025-01-23

**Authors:** Nur Adila Mohd Norwir, Shahida Mohd-Said, Aznida Firzah Abdul Aziz, Tuti Ningseh Mohd-Dom

**Affiliations:** 1 Oral Health Programme, Ministry of Health, Putrajaya 62590, Malaysia; drdrnuradila@moh.gov.my; 2 Faculty of Dentistry, Universiti Kebangsaan Malaysia, Kuala Lumpur 50300, Malaysia; tutinin@ukm.edu.my; 3 Faculty of Medicine, Universiti Kebangsaan Malaysia, Kuala Lumpur 50300, Malaysia; draznida@hctm.edu.my; 4 The Family Oral Wellness Research Group, Universiti Kebangsaan Malaysia, Kuala Lumpur 50300, Malaysia

**Keywords:** integrated care, patient compliance, primary healthcare, referral and consultation, risk assessment

## Abstract

**Background/Objectives**: Opportunistic diabetes screening in dental clinics is an innovative strategy with significant public health implications. **Methods**: This prospective observational study assessed diabetes risk and referral compliance among periodontitis patients using the Finnish Diabetes Risk Score (FINDRISC) and capillary fasting blood glucose (cFBG). Patients with FINDRISC ≥ 11 and/or cFBG ≥ 5.6 mmol/L were classified as high-risk and referred for further medical evaluation, with compliance tracked through medical practitioner feedback. **Results**: A total of 142 participants were recruited by 20 general dental practitioners (GDPs). Of these, 36.4% (n = 47) had a FINDRISC ≥ 11, with a mean score of 7.7 ± 4.5, and 26.3% (n = 34/129) had cFBG levels ≥ 5.6 mmol/L. There was no significant difference between periodontal status and FINDRISC (*p* = 0.291) or between periodontal status and cFBG (*p* = 0.129). Overall, 54 patients (41.8%) were referred for follow-up, with 33 (61.1%) completing the process. Among those who completed referrals, 10 (30.3%) were diagnosed with prediabetes and seven (21.2%) with diabetes. Non-compliance was more common among patients from lower socioeconomic backgrounds. **Conclusions**: This study shows the feasibility of integrating diabetes risk screening into private dental practice and its potential to identify high-risk individuals. Shared care models and policy adaptations are essential to improve interdisciplinary collaboration and overcome referral compliance barriers.

## 1. Introduction

The connection between periodontal diseases and diabetes is well-established, with each condition exacerbating the other and complicating treatment outcomes [1]. Diabetes increases the risk of periodontitis by two to three times, while poor periodontal health raises the likelihood of developing diabetes [2,3]. This bidirectional relationship emphasises the potential for dental clinics to play a critical role in opportunistic diabetes screenings [4]. Numerous studies suggest that such screenings effectively identify at-risk patients who may not seek regular medical care, enabling early intervention and the management of both conditions [5,6,7].

Periodontal treatment significantly benefits diabetic patients by lowering HbA1c levels. HbA1c reductions of 0.3 to 0.4% has been shown within three to four months post-treatment of non-surgical periodontal therapy (NSPT) [2]. Reduction of HbA1c of 0.47% to 0.6% was observed in groups receiving intensive periodontal therapy versus the group receiving control periodontal treatment such as scaling and OHI only [8,9]. NSPT alone has been reported to be very effective in reducing periodontal inflammation [10]. Additionally, a meta-analysis suggested that periodontal treatment of T2DM patients resulted in a decrease of 0.4% in HBA1c level following periodontal treatment [11]. These indicated that treatment of periodontitis could be important for effective management of diabetes.

Interleukins (ILs) are key cytokines that regulate immune responses and inflammation, playing an important role in both Type 1 Diabetes (T1DM) and periodontitis. In T1DM, pro-inflammatory cytokines such as IL-1β, IL-2, IL-12, and IL-6 are elevated, contributing to the autoimmune destruction of pancreatic beta cells and driving disease progression. These same interleukins are also implicated in periodontitis, where they aggravate both local gum inflammation and systemic inflammation, potentially worsening diabetes control [10,12]. This has led to suggestions that ILs could be a potential target for the development of new treatments for the disease.

Incorporating diabetes screenings into dental practices not only addresses immediate health concerns but also enhances patient education about the interplay between oral and systemic health [13]. Equipping dental professionals with screening tools allows them to identify individuals at high risk for both conditions [14,15], thereby enabling proactive management strategies that extend beyond traditional dental care. This integrated approach improves patient compliance and engagement, as those who understand the connection between their oral health and chronic diseases are more likely to seek comprehensive care [16,17]. The association between oral health and systemic diseases, including periodontitis’ link to coronary heart disease and myocardial infarction, is well-documented. Integrating oral health assessments into routine medical evaluations facilitates early disease detection and empowers patients to make informed decisions about their health [18].

Global recommendations support such integration. The Joint European Federation of Periodontology (EFP)/American Association of Periodontology (AAP) Workshop on Periodontitis and Systemic Diseases emphasised the importance of managing periodontitis patients at risk of or with diabetes [19]. In fact, the International Diabetes Federation (IDF) had also recognised that dentists should use screening tools to assess diabetes risk and refer patients to physicians for diagnostic testing and management [20]. Patients with diabetes risk factors should be informed of the risk and referred for diagnostic testing and follow-up care [21]. In Malaysia, the Ministry of Health has included dental referrals in its Clinical Practice Guidelines for Type 2 Diabetes Management, highlighting the importance of interdisciplinary collaboration [22]. The pathway for the dental referral had been deliberated by a multidisciplinary team to guide dentists on the protocol for screening and referring diabetes-at-risk patients [23].

Despite these recommendations, the uptake of referral protocols remains unknown. The alarming prevalence of diabetes in Malaysia further highlights the urgent need for actionable strategies to improve early detection rates [24]. This growing health crisis poses significant challenges to the healthcare system and highlights the need for proactive measures to identify at-risk individuals before the disease progresses [25]. Studies have shown that patients are generally accepting of medical screenings conducted within dental clinics, which is crucial for the successful implementation of this strategy [26], and the majority of dentists are willing to screen and refer patients for diabetes management by medical doctors [27].

This study aims to evaluate the feasibility of identifying diabetes risk among periodontitis patients in Malaysian private dental clinics and to assess their compliance with referrals for medical care. The findings could provide valuable insights for enhancing referral practices and advancing a more integrated healthcare approach to improve patient outcomes.

## 2. Materials and Methods

Ethics approval for the study was granted by the Research Committee Board in October 2020 (PPl/111/8IJEP-2020-617). This study was funded by a grant from the Ministry of Higher Education (MRUN-RAKAN RU-2019-002/2) and registered under research code (DD-2020-03).

### 2.1. Study Design

**Figure 1:**
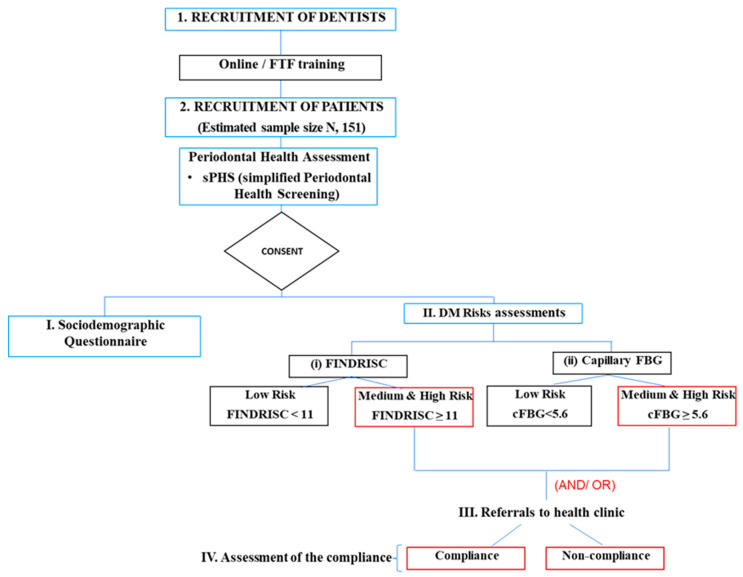
Study Flow.

This study was designed as a prospective observational study with a two-stage management protocol: (i) specific diabetes risk identifications and (ii) referral of at-risk patients to health clinics for diabetes evaluation (Figure 1). The first stage involved screening participants using established risk assessment tools, while the second stage focused on ensuring timely follow-up and support for those referred to specialised care. This study’s recruitment and follow-up period was from October 2020 until March 2022 (18 months). The study was executed in four phases: (i) initial intra-oral examination (ii) diabetes risk and glycaemic level assessment (iii) referral for medical assessment and (iv) compliance assessment at dental clinic.

### 2.2. Recruitment of Participating Dentists

Eligible general dental practitioners (GDPs) from the researchers’ network were individually invited to participate in the study. To be eligible, GDPs had to meet the inclusion criteria (Table 1). All participating GDPs underwent specialised training provided by the principal investigator (PI), focusing on periodontitis and diabetes screenings to ensure standardisation. GDPs were selected from private practices for their ability to offer immediate follow-up care and treatment. In Malaysia, general dentists are trained to refer cases requiring specialist management to periodontists. Participation was voluntary, with no incentives offered.

### 2.3. Recruitment of Participating Patients

Participating GDPs evaluated potential patients at their practices and encouraged participation. Patients were required to meet specific inclusion and exclusion criteria (Table 2). Consenting patients received study information and a consent form and filled out a questionnaire on demographics and medical history.

### 2.4. Screening for Periodontal Disease Classification

We used a simplified digital periodontal health screening module according to the simplified Periodontal Health Screening tool [28], designed to assist GDPs in efficiently screening patients’ periodontal health. The module includes history taking, clinical assessment, and case decision, following guidelines from the British Society of Periodontology’s Basic Periodontal Examination (BSP 2019), Periodontal Risk Assessment (PRA), periodontitis self-assessment, and the 2017 Classification of Periodontal and Implant Diseases. GDPs received training sessions to standardise parameter use, disease classification, and reporting.

### 2.5. Diabetes Risk Assessment

We conducted the diabetes risk assessment using the well-established Finnish Diabetes Risk Score (FINDRISC) screening tool [29]. FINDRISC includes eight inquiries: age, body mass index (BMI), waist circumference (WC), intake of blood pressure medication, past occurrences of elevated blood glucose, levels of physical activity, daily intake of vegetables, fruits or berries, and family history of diabetes. Each response to the questions was assigned a different weighted score, culminating in a maximum possible score of 26. Based on the risk score, patients were categorised accordingly into low-risk, elevated-risk, moderate-risk, high-risk, and very high-risk classifications. In this study, we used the proposed ideal FINDRISC cut-off value for undiagnosed diabetes of scores ≥11, which requires a referral to health clinics for additional diabetes assessment [30].

We included capillary Fasting Blood Glucose (cFBG) testing for an objective assessment of blood glucose levels, enhancing risk identification by complementing FINDRISC. Patients were instructed not to eat for at least 8 h before their dental appointment. On the evaluation day, a finger-prick blood sample was taken and cFBG levels measured with an Accu-Chek^®^ glucometer (Roche Diabetes Care, Basel, Switzerland). A fasting blood glucose level over 5.6 mmol/L was considered elevated, per the Malaysian Clinical Practice Guidelines for Managing Type 2 Diabetes Mellitus (T2DM) {22].

### 2.6. Referral and Compliance Tracking

Patients at risk for diabetes (FINDRISC score of 11 or higher and/or cFBG levels over 5.6 mmol/L) were referred to a healthcare facility for further evaluation. They received referral documents and were asked to return with the completed form within four months. Reminders were sent at two months. Patients who did not submit the form after four months were considered non-compliant.

### 2.7. Sample Size Calculation

This study used non-probability sampling. The sample size was calculated based on Malaysia’s undiagnosed diabetes rate [31,32]. With the highest undiagnosed rate at 12.1% [32] and a 10% dropout rate included, the minimum required sample size was 151 patients.

### 2.8. Statistical Analysis

The analysis used IBM SPSS version 26.0 for both descriptive and inferential statistics. Descriptive statistics summarised mean ± standard deviation (SD) and percentage for demographics, diabetes risks, and glycaemic levels. Pearson’s Chi-squared test assessed relationships between categorical variables, and Fisher’s Exact Test analysed associations when expected cell counts were below 5. One-way ANOVA explored the link between sociodemographic factors and diabetes risks in periodontitis patients, with a 95% confidence level (α = 0.05) set for statistical significance.

## 3. Results

### 3.1. General Dental Practitioners’ Recruitment

Using convenience sampling, 62 invitations were sent to GDPs. Of these, 23 (37.1%) agreed to participate, 28 (45.2%) declined, and 11 (17.7%) did not respond. However, three GDPs who consented and were trained did not enroll their patients during data collection.

### 3.2. Patients’ Demographics and Periodontal Status

A total of 158 individuals were screened for the study, with 16 declining participations. The remaining 142 participants, aged 19–74 (mean age 42.2 ± 12.7), included 62 males (42.3%). Over half (57.7%) had ’College or University’ education. Socio-economically, 54.2% were low-income, 37.3% middle-income, and the rest high-income. Medical history showed that 33.1% had non-communicable diseases like hypertension, cardiovascular diseases (CVD), and dyslipidaemia. With regards to their periodontal health, 30 patients (21.1%) were classified as Periodontitis Stable, 43 patients (30.3%) as Periodontitis in Remission, and 69 patients (48.6%) as Periodontitis Unstable (Table 3). 

### 3.3. Diabetes Risk and Glycaemic Level

Based on the FINDRISC screening results, two (1.4%) patients were classified as Very High-Risk for undiagnosed diabetes, eight (5.6%) fell into the High-Risk category, 21 (14.8%) were categorised as Moderate-Risk, 46 (32.4%) were in the Elevated group, and 65 (45.8%) patients were placed in the Low-Risk group (Table 4).

Regarding (cFBG), 95 participants (73.6%) had cFBG levels at or below 5.6 mmol/L, 26 participants (20.2%) fell within the prediabetic range, and the remaining eight participants (6.2%) exhibited cFBG levels at or above 7.0 mmol/L (Table 5). In all, 34 (26.4%) patients demonstrated cFBG ≥ 5.6 mmol/L.

### 3.4. Periodontitis Status and Diabetes Risk

**Figure 2:**
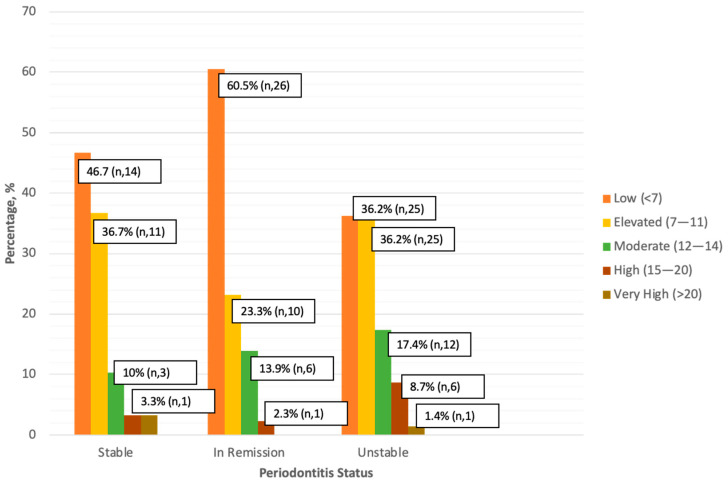
Diabetes Risk Assessment in Periodontitis Patients based on FINDRISC (n, 142).

All periodontitis status groups showed similar risk patterns, with most patients in the low-risk category and the fewest in the higher-risk category (Figure 2). For stable periodontitis, 46.7% were at low risk and 36.7% at elevated risk for diabetes. For periodontitis in remission, 60.5% were at low risk and 23.3% at elevated risk. Highest percentage of patients in medium, high, and very high FINDRISC score groups were patients with periodontitis unstable (i.e., 17.4%, 8.7%, and 1.4% respectively). However, no significant difference was observed between FINDRISC score groups and periodontitis status (Fisher’s exact test, *p* = 0.291).

### 3.5. Periodontitis Status and Glycaemic Level

**Figure 3:**
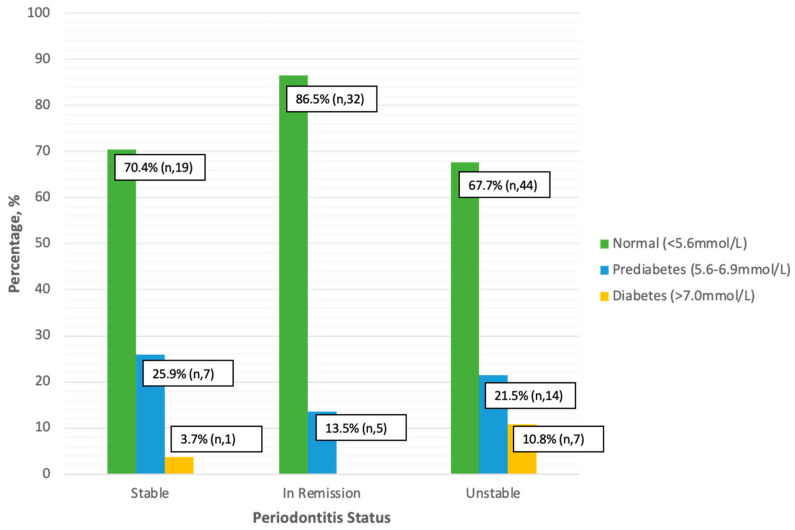
Diabetes Risk Assessment in Periodontitis Patients based on cFBG (n, 129).

Many subjects had normative cFBG levels, regardless of their periodontitis status (Figure 3). A higher percentage of prediabetic (10.9%) and diabetic (5.4%) individuals had unstable periodontitis, but this difference was not statistically significant (Fisher’s Exact Test, *p* = 0.129).

### 3.6. Patients Requiring Referral for Further Diabetes Assessment

Table 6 summarises the correlation between diabetes risk categories, assessed using the FINDRISC, and glycaemic levels, measured through cFBG. Of the 129 participants assessed, 73.6% had normal glycaemic levels, 20.2% showed prediabetes, and 6.2% were diagnosed with diabetes. Most patients with low FINDRISC scores were detected with normal readings of cFBG (i.e., 84.7%), while patients with higher scores had generally higher cFBG readings. A statistically significant relationship was observed between FINDRISC risk categories and glycaemic levels (Fisher’s Exact test, *p* = 0.001), indicating that higher FINDRISC scores are associated with a greater likelihood of prediabetes or diabetes.

Table 7 presents the number of patients needing referrals for further medical assessment based on FINDRISC scores and cFBG readings. The table indicates that 45 patients require referrals due to an increased FINDRISC score. An additional nine patients need medical assessment due to their prediabetes and diabetes status, as identified by elevated cFBG scores. Therefore, a total of 54 out of the 129 participants require further medical assessment.

Among patients requiring referrals, the highest percentage of those needing further diabetes assessment were patients with unstable periodontitis (Table 8). There was a statistically significant association between periodontitis status and the need for referral to a health clinic (Chi square test, *p* = 0.012).

### 3.7. Compliance to Referral

Of 54 patients referred to medical practitioners for further diabetes evaluation, 33 (61.1%) patients attended their referral. All reported to have received a diabetes assessment, including an HbA1c test as a standard protocol for diagnosing diabetes mellitus, i.e., T2DM (>45 mmol/mol), prediabetes (29–44 mmol/mol), and normal (<39 mmol/mol) (Ministry of Health, 2015). Based on the medical practitioner’s assessment, the confirmatory diagnosis of 33 periodontitis patients that attended the referral was as follows: 16 (48.5%) were non-diabetic, 10 (30.3%) were prediabetic, and seven (21.2%) were diabetic.

## 4. Discussion

### 4.1. Feasibility of Diabetes Risk Screening in Dental Clinics

Diabetes risk screening in dental clinics is becoming a viable method for identifying individuals at risk of undiagnosed diabetes [4,5,7]. This study shows that using the (FINDRISC) along with (cFBG) tests in private dental clinics could be integrated into routine dental care. FINDRISC offers a simple, non-invasive way to assess diabetes risk, while cFBG provides an immediate measure of glycaemic levels for accurate risk identification. Previous research supports the effectiveness and patient acceptance of similar screenings in dental settings [5,26]. However, only one-third of the dentists who were approached agreed to participate. This reluctance may stem from time constraints, lack of training or confidence in diabetes risk assessments, perceived irrelevance to their dental practice, and concerns about patient reactions to systemic screenings in dental settings [27]. Addressing these barriers through training, better integration of tools, and clear communication about the benefits of interdisciplinary care could enhance participation.

### 4.2. High Prevalence of Undiagnosed Risk

The high prevalence of undiagnosed diabetes risk observed in this study confirms the hidden burden of diabetes among periodontitis patients. Approximately 26.4% of participants exhibited abnormal glycaemic levels, classified as prediabetes or diabetes, while 27 patients had moderate to very high FINDRISC scores. This is consistent with findings from similar studies, which have reported elevated rates of undiagnosed diabetes and prediabetes in dental settings, particularly among individuals with periodontal disease [5,7]. Such results reinforce the role of dental clinics as a valuable point of care for opportunistic screening, enabling early detection of at-risk individuals who might otherwise remain undiagnosed. However, challenges remain, including ensuring follow-up care for identified patients and addressing the potential underestimation of risk in individuals with normal cFBG but elevated FINDRISC scores. Additionally, while this study highlights the prevalence of undiagnosed risk in a private dental clinic setting, findings may not be fully generalisable to populations in public or rural healthcare settings, where access to care and health literacy may differ.

### 4.3. Correlation Between Periodontitis and Diabetes Risk

Although no correlation was found between periodontal status and FINDRISC or cFBG in this study, other research supports this link. For example, a study on patients with severe periodontitis (Stages III and IV) found that 75% of those with a FINDRISC ≥ 12 had an increased risk for diabetes (HbA1c ≥ 5.7%) [33]. This could be due to the inclusion of patients with prediabetes or diabetes at baseline. Another study showed that clinical periodontal measurements, such as missing teeth and pocket probing depth ≥6 mm, can help identify diabetic patients and improve FINDRISC’s accuracy as a diabetes screening tool [34].

Patients with unstable periodontitis showed higher FINDRISC scores and cFBG levels, suggesting a link between poor periodontal health and increased diabetes risk. While the observed associations lacked statistical significance in some cases, the findings align with global literature demonstrating the bidirectional relationship between periodontitis and diabetes. Chronic inflammation caused by periodontitis is known to exacerbate systemic insulin resistance, contributing to hyperglycaemia, while poorly controlled diabetes increases susceptibility to periodontal infections [2,21]. However, variations in study populations and methodologies may influence the strength of these associations. This study probably lacked power because the final sample size was less than the projected required number.

### 4.4. Barriers to Compliance

The compliance rate, which was 61% of referred periodontitis patients with increased diabetes risk attending the health clinic in this study, is encouraging. In the past, mixed reports of referral compliance ranging from 20% to as high as 80% to 88% have been reported in the literature [28,35,36,37]. Although 61.1% of patients referred for further diabetes evaluation complied with their referrals, nearly 39% failed to attend follow-ups, suggesting significant gaps in care continuity.

A study on the determinants of non-compliance with physician referrals identified several barriers, including limited awareness of health risks after screening, poor self-management habits, fear of diagnosis and healthcare costs, optimistic beliefs stemming from the absence of symptoms, competing social obligations, aversion to medication, and difficulties with communication and scheduling follow-up appointments [38]. Interventions such as providing financial subsidies for diagnostic testing, leveraging telemedicine to simplify follow-ups, and integrating motivational interviewing during dental visits may help address these barriers. Future efforts should focus on patient-centred strategies to improve compliance and ensure timely medical follow-up for at-risk individuals.

### 4.5. Role of Interdisciplinary Care

This study emphasises the need for a shared care approach between dental and medical practitioners to manage patients with interconnected systemic and oral health conditions. By integrating structured referral pathways and strengthening communication between healthcare providers, interdisciplinary collaboration can enhance the early detection and management of conditions like diabetes among periodontitis patients. Evidence supports that interdisciplinary care improves patient outcomes, reduces healthcare fragmentation, and addresses comorbidities holistically [7,13,39]. However, the feasibility of implementing such models requires addressing challenges like inadequate training in systemic health for dental professionals and limited cross-disciplinary interaction. Available national guidelines for shared care protocols must be translated into action. This can be achieved by providing continuous education to practitioners, which would strengthen this approach and promote collaboration across healthcare domains.

### 4.6. Policy Implications

The study highlights the need for policy adaptations to integrate diabetes screening into standard dental practice. Key policy changes could include mandatory training programmes for dental practitioners to enhance their skills in identifying systemic health risks, subsidising the cost of chairside screening tools to ensure accessibility across private and public dental clinics, and launching nationwide campaigns to increase public awareness about the relationship between oral and systemic health. Adopting interdisciplinary management for patients at risk of diabetes within Malaysia’s healthcare framework could foster a more integrated approach to managing chronic conditions and improve early detection and intervention efforts. However, effective implementation will require stakeholder engagement, robust infrastructure, and adequate funding to support these initiatives.

### 4.7. Public Health Impact

This study highlights the potential public health impact of incorporating diabetes screening into routine dental visits. By identifying high-risk individuals early, this approach could help reduce the progression and complications of diabetes, particularly in high-prevalence countries like Malaysia. Early detection via dental screening could lead to better health outcomes and lower healthcare costs. Integrating oral and systemic health initiatives supports holistic patient care and aligns with the National Oral Health Strategic Plan 2022–2030, which focuses on strengthening primary care services. Implementing this screening programme will require training dental practitioners, providing necessary resources, and fostering collaboration between dental and medical professionals. Overcoming these challenges could enhance Malaysia’s healthcare system or other countries who face similar situations.

### 4.8. Limitations and Areas for Future Research

While this study provides valuable insights, it is not without limitations. The reliance on convenience sampling may limit the generalisability of findings, as participants were primarily drawn from private dental clinics, potentially excluding underserved populations in public or rural settings. Next, the absence of a calibration exercise for participating dental practitioners may have impacted the consistency and accuracy of diagnosing and classifying periodontitis. Variations in clinical judgment could influence the reliability of findings related to periodontitis status and its association with diabetes risk. Addressing this issue in future studies would enhance diagnostic standardisation and ensure more robust data collection. Another limitation is the lack of clinical periodontal parameters, such as bacterial plaque. Examining these factors could provide further insight into their association with other study variables.

Future research should explore the cost-effectiveness of integrating diabetes screening into dental practices, as economic feasibility is critical for widespread adoption. Long-term studies tracking patient outcomes and assessing the impact of early detection on healthcare systems are also necessary. Furthermore, extending this screening model to public healthcare settings and rural populations could provide a more comprehensive understanding of its scalability, accessibility, and effectiveness in addressing health disparities.

## 5. Conclusions

This study demonstrates the feasibility of integrating diabetes risk screening into dental practice. Key findings showed 41.8% percent of periodontitis patients to be at risk for hyperglycaemia, and one-third of the referred patients were confirmed with prediabetes or diabetes. Compliance rate of periodontitis patients referred to health clinics with increased diabetes risk is high. Barriers like socioeconomic challenges and referral issues can be addressed by integrating diabetes screening, training dental practitioners, and offering support. Leveraging routine dental visits for early detection can reduce undiagnosed diabetes and improve health outcomes in Malaysia.

## Figures and Tables

**Table 1:** Inclusion and Exclusion Criteria for Participating Dentists.

Inclusion	Exclusion
Registered with the Malaysian Dental Council (MDC) Holds a valid Annual Practicing Certificate (APC)	Less than 2 years of professional experience

**Table 2:** Inclusion and Exclusion Criteria for Participating Patients.

Inclusion	Exclusion
Diagnosed with periodontitis	Inability to read, write, or understand Malay or English
18 years or older	Expectant mothers
Malaysian citizens	
No diabetes mellitus diagnosis	

**Table 3:** Patients’ Demographics and Periodontal Status.

Demographic	Number	Percentage
Age (mean years, SD)	42.23 (±12.71)	-
Gender		
Male	62	42.3
Female	80	57.7
Education level		
No formal	-	
Primary	5	3.5
Secondary	55	38.7
College/University	82	57.7
Monthly Household Income		
(a) Low income B40 (<RM4800)	77	54.2
(b) Middle income M40 (RM4800–RM10980)	53	37.3
(c) High income T20 (≥RM10980)	12	8.5
Comorbidities		
Yes	47	33.1
No	95	66.9
Periodontitis Current Health Status		
Currently Stable	30	21.2
Currently In Remission	43	30.3
Currently Unstable	69	48.7

B40: Low-income group (household income less than RM4800). M40: Middle-income group (household income between RM4800 and RM10980). T20: High-income group (household income greater than or equal to RM10980). SD: Standard Deviation.

**Table 4:** Diabetes Risk Screening.

FINDRISC, n (%)					
Low (<7)	Elevated (7–11)	Moderate (12–14)	High (15–20)	Very High (>20)	Total
65 (45.8)	46 (32.4)	21 (14.8)	8 (5.6)	2 (1.4)	142 (100)

**Table 5:** Glycaemic Level.

Glycaemic Level (cFBG, mmol/L)			
Normal n (%)	Prediabetes n (%)	Diabetes n (%)	Total n (%)
95 (73.6)	26 (20.2)	8 (6.2)	129 (100)

**Table 6:** Correlation between FINDRISC and cFBG.

FINDRISC	Glycaemic Level (cFBG, mmol/L)			Total	*p*-Value
	Normal	Prediabetes	Diabetes		
Low	50 (84.7)	7 (11.86)	2 (33.9)	59 (100)	* 0.001
Elevated	33 (76.7)	9 (20.9)	1 (2.3)	43 (100)	
Medium	9 (50.0)	6 (33.3)	3 (16.7)	18 (100)	
High	3 (42.9)	2 (28.5)	2 (28.5)	7 (100)	
Very High	0	2 (100)	0	2 (100)	
Total	95 (73.6)	26 (20.2)	8 (6.2)	129 (100)	

* significant at *p* < 0.05.

**Table 7:** Patients Requiring Referral for Medical Assessment.

FINDRISC	Glycaemic Level (cFBG, mmol/L)			
	Normal	Prediabetes	Diabetes	Total n (%)
<11	75	7	2	84 (65.1)
≥11	20	* 19	* 6	45 (34.9)
Total	95 (73.6)	26 (20.2)	8 (6.2)	129 (100)

* patients referred based on both elevated cFBG and increased FINDRISC score

**Table 8:** Periodontal status of patients requiring medical referral.

Periodontitis Status	Require Referral			
	Yes	No	Total	*p*-Value
Stable	10 (18.5%)	17 (22.7%)	27 (20.9%)	* 0.012
In Remission	9 (16.7%)	28 (37.3%)	37 (28.7%)	
Unstable	35 (64.8%)	30 (40%)	65 (50.4%)	
Total	54	75	129	

* significant at *p* < 0.05.

## Data Availability

The raw data supporting the conclusions of this article will be made available by the authors on request.
